# The Effects of Daily Sleep Condition on Performances of Physical Fitness among Taiwanese Adults: A Cross-Sectional Study

**DOI:** 10.3390/ijerph17061907

**Published:** 2020-03-15

**Authors:** Chi-Chieh Hsu, Ming Gu, Tian-Shyug Lee, Chi-Jie Lu

**Affiliations:** 1 Department of Aquatic Sports, University of Taipei, Taipei City 11153, Taiwan; markcchsu@gmail.com; 2 Graduate Institute of Business Administration, Fu Jen Catholic University, New Taipei City 242304, Taiwan; gm19850617@gmail.com (M.G.); 036665@mail.fju.edu.tw (T.-S.L.); 3 Artificial Intelligence Development Center, Fu Jen Catholic University, New Taipei City 242304, Taiwan; 4 Department of Information Management, Fu Jen Catholic University, New Taipei City 242304, Taiwan

**Keywords:** Sleep duration, Sleep quality, BMI, Sit-ups, Sit-and-reach, CEI, MARS

## Abstract

Physical fitness is a powerful indicator of health. Sleep condition plays an essential role in maintaining quality of life and is an important marker that predicts physical fitness. This study aimed to determine the relationship between sleep conditions (sleep quality, sleep duration, bedtime) and multiple physical fitness indicators (body mass index (BMI), flexibility, abdominal muscle strength and endurance, cardiopulmonary endurance) in a well-characterized population of Taiwanese adults aged 23 to 65. The applied data were obtained from the National Physical Fitness Examination Survey 2014 conducted in Taiwan. We assessed the association of the sleep conditions with physical fitness performances in Taiwanese adults by using the multivariate adaptive regression spline (MARS) method with a total of 69,559 samples. The results show that sleep duration, sleep quality, and bedtime were statistically significant influence factors on physical fitness performances with different degrees. Gender was an important factor that affects the effects of daily sleep conditions on performances of physical fitness. Sleep duration was the most important factor as it was simultaneously correlated with BMI, sit-ups, and sit-and-reach indicators in both genders. Bedtime and sleep quality were only associated with sit-ups performance in both genders.

## 1. Introduction

Sleep condition is an important predictor of health status. Many studies have explored the relationship between sleep conditions and health responses. For example, research has determined that short and long sleep durations were significantly associated with poor self-rated health and lower quality of life [1]. Sleep quality was shown to have a significant association with the poor quality of life of patients with diseases [2,3,4,5,6]. Individuals with inappropriate sleep duration had an increased risk of all-cause mortality, cardiovascular disease, and developing symptomatic diabetes [7].

Physical fitness is a powerful indicator of health and should be a major goal in current and future public health promotion policies [8]. It comprises a set of important attributes that are either health- or skill-related and can be objectively measured through functional fitness [9]. The quantification of physical fitness is mainly evaluated by the morphological component (overweight status), the muscular component (endurance, power fitness), the motor component (coordination) and the cardio-respiratory component (cardiovascular systems) [10,11,12,13,14]. The latest development with regard to physical fitness and several health outcomes suggested that physical fitness is associated with total and abdominal adiposity, cardiovascular disease, muscular fitness and speed/agility, skeletal health, quality of life, depression, anxiety, and mood status [15,16,17,18,19,20,21].

Many studies have explored the relationship between lifestyle and physical fitness, as it provides useful information for designing effective health improvement programs. For example, Liao et al. (2016) showed that sedentary behavior and daily traveling by private motorized vehicle was associated with poor performance in abdominal muscular strength and endurance [22]. Cabanas-Sánchez et al. (2018) found that sedentary time, diet quality, and physical activity influence children’s physical fitness performance [23]. Ruedl et al. (2019) studied the influences of lifestyle habits such as weekly leisure time, sports activities and daily electronic media usage on physical fitness. They suggested that more leisure time sports activity and lower daily electronic media usage can increase physical fitness in youth [24]. A study by Alghadir et al. (2020) found that sitting time associated with media use influences physical fitness. However, relatively few studies have focused on the effect of sleep conditions on physical fitness performance and most of the existing studies were aimed at skill-related physical fitness [25]. Bulbulian et al. (1996) found the negative effect of sleep deprivation on leg strength and endurance [26]. The prevalence of sarcopenia appears also to be associated with sleep quality [27]. Beccuti and Pannain (2011) showed that sleep restriction leads to hormonal alterations, which may favor an increase in calorie intake and a decreased energy expenditure, and ultimately leads to BMI rise and weight gain [28]. Garaulet et al. (2011) pointed out that in European adolescents, especially females, short sleep duration was significantly associated with higher values of body mass index (BMI) [29].

The accurate quantification of physical fitness is essential to evaluate health outcomes and the effectiveness of intervention programs [30]. The Sports Administration, Ministry of Education in Taiwan (MOE-SA) assessed the four core physical fitness indicators through the measurements of BMI, bent-leg sit-up test, sit-and-reach test, and 3-min step test. The measurement of BMI reflects the probability of being overweight; the bent-leg sit-up test reflects lumbar and muscle endurance; the sit-and-reach test reflects hamstring flexibility and also examines tension of the movable range of the lower back joint or the toughness of the ligament; and the 3-min step test reflects cardiorespiratory endurance index (CEI) [22]. Once a year, MOE-SA conducts such a survey called the National Physical Fitness Survey (NPFES). The current study used data from the NPFES and an effective data mining method—multivariate adaptive regression spline—to explore the effects of sleep conditions (sleep quality, sleep duration and bedtime) on four core physical fitness indicators.

## 2. Methods

### 2.1. Study Design

The NPFES is a cross-sectional assessment conducted by MOE-SA to investigate physical fitness performance. Participants were recruited using convenience sampling at 35 test stations in 22 cities and counties in Taiwan. The physical fitness outcomes (BMI, sit-ups, sit-and-reach, CEI) were collected and a standardized face-to-face interview was conducted using a structured questionnaire which contained sleep condition variables. The questionnaire design and survey process was uniformly set and implemented by MOE-SA. The survey was approved by the MOE-SA and written informed consent was obtained from each participant. Both the investigators and the participants understood the investigation process prior to the survey. The computerized data set of the NPFES released by the Sports and Health Information Application Research Center of the MOE-SA for public research purposes contained information on the demographic characteristics, lifestyle behavior, and physical fitness measurements of each participant. These data comprised de-identified secondary data, which were released to the public for research purposes [22]. This study was approved by the Institutional Review Board of Fu Jen Catholic University in Taiwan.

### 2.2. Testing Procedures

All physical fitness tests were measured by trained examiners. A standardized training instruction manual, which included detailed procedures for measuring various physical fitness indicators, was provided to trained examiners who conducted the fitness tests. BMI was calculated as body weight divided by height squared. Body height and weight were measured using wall-mounted tape measures and electronic scales. The measurement unit of body height is cm and that of body weight is kg. The abdominal muscular strength and endurance was measured using a timed bent-leg sit-up test. The unit of sit-ups is frequency/min. Flexibility was measured using a modified sit-and-reach test, which was considered a useful field test to evaluate hamstring and low-back flexibility. The measurement unit of sit-and-reach test is cm. The cardiorespiratory endurance was measured using an adapted 3-min step test from the Harvard Step Test, which involved repeatedly stepping on and off a 35-cm step for 3 min. The cardiorespiratory endurance index (CEI) was used as an indicator of cardiorespiratory fitness performance and calculated using the post exercise recovery heartbeat counts in intervals of 1 to 1.5 min, 2 to 2.5 min, and 3 to 3.5min.

In addition, after the physical fitness test, a standardized questionnaire of NPFES for demographic and behavioral items including questions about sleep conditions was provided to the participants by trained interviewers, and each participant answered the questionnaire voluntarily. The question about sleep duration included in the questionnaire was the self-assessed average daily sleep duration. Similarly, the question about the bedtime in the questionnaire was the average daily bedtime that the subjects were also asked to self-assess. The question about sleep quality used a 4-point Likert scale to query self-assessed sleep quality. The principle of the score was the number of days when the respondent found it difficult to fall asleep in a week: answer 1 means no or very few (less than 1 day); 2 means sometimes (1–2 days); 3 represents often (3–4 days); 4 represents always (5–7 days).

### 2.3. Participants

A total of 69,559 Taiwanese adults aged 23 to 65 years were recruited to participate in the 2014 National Physical Fitness Survey (NPFES 2014). NPFES is conducted once a year, and the content of the questionnaire is slightly different each year. We selected the data of NPFES 2014 for analysis as it contained the most sleep condition variables (sleep quality, sleep duration, bedtime) and physical fitness variables (measurements of BMI, sit-and-reach test, bent-leg sit-up test and 3-min step test). After the data cleaning process, through deleting invalid questionnaires, missing values and outlier interpolation, we obtained 62,094 valid data samples.

### 2.4. Statistical Analysis

We used the ‘earth’ package of version 5.1.1 proposed by [31] in R software of version 3.5.3 (R core team, Vienna, Austria) for multivariate adaptive regression spline (MARS) model to analyze the effects of sleep conditions on physical fitness indicators. MARS is an effective and promising non-parametric data mining method and uses a specific class of base functions, known as “knot” predictors [32]. A knot marks the end of one region of data and the beginning of another [33]. Variables, i.e., gender, age, sleep duration, sleep quality, and bedtime, were all placed in the corresponding basis function to get the trend of knot position and segment estimation. The MARS procedure is divided into three steps. Firstly, a forward algorithm was applied to select all possible basic functions and their corresponding knots. Next, in order to generate the best combination of existing knots, a backward algorithm was used to eliminate all basic functions in the order of least contributions based on the generalized cross-validation (GCV) criterion. GCV is a form of regularization and trades off goodness-of-fit against model complexity. The best combination was the one with the lowest GCV value. Finally, a smoothing operation was performed to obtain continuous partition borders [34]. Through the MARS method, we determined whether each independent variable was included in the estimation equation and got the best segmentation estimation results. The coefficient corresponding to the variable represents the trend and extent of the influence of the independent variable on the dependent variable. The regression coefficient estimation and significance determination of the model were obtained by selecting significant influence variables from the set of variables and implemented piecewise estimation. The p-value of the estimated regression coefficient for significance was set to 0.05.

For correlation analysis, the ‘psych’ package of version 1.9.12.31 proposed by [35] in R software of version 3.5.3 (R core team, Vienna, Austria) was applied to produce the correlation coefficients between sleep conditions and physical fitness indicators. Spearman’s rank correlation coefficient is appropriate when several variables are ordinal. The rule of thumb for interpreting the size of a correlation coefficient result is as follows: when the correlation coefficient falls within the interval of 0.90 to 1.00 (−0.90 to −1.00), it can be interpreted as a very high positive (negative) correlation; and the interval 0.70 to 0.90 (−0.70 to −0.90) stands for a high positive (negative) correlation; the interval 0.50 to 0.70 (−0.50 to −0.70) represents moderate positive (negative) correlation; the interval 0.30 to 0.50 (−0.30 to −0.50) signifies a low positive (negative) correlation; the interval 0.00 to 0.30 (0.00 to −0.30) means a negligible correlation [36]. The *p*-value of the correlation coefficient estimate for significance was set to 0.05.

## 3. Results

The statistical description results of the used data are shown in Table 1. From the table, we can see that BMI ranged from 13.67 to 68.13 (mean 23.88), sit-ups ranged from 0 to 80 (mean 23.20), sit-and-reach ranged from −4 to 70 (mean 24.73), and CEI ranged from 11.78 to 155.17 (mean 57.32). Variables were organized according to gender (male = 0, female =1), age, sleep quality level (1 to 4, with a higher level representing worse sleep quality), sleep duration, and the converted bedtime variable (0 = 12 midnight). Age and sleep duration variables are natural values. It also can be seen from Table 1 that, based on gender, 29,562 (48%) subjects were men, and 32,532 (52%) subjects were women. Age ranged from 23 to 64, with a mean of 41.24 years old. Sleep quality level ranged from 1 to 4 with a median level of 2. Sleep duration ranged from 4 to 13 hours, with median of 7 hours. For the sake of further data analysis, we transformed the original 24-hour clock system of the bedtime variable to a transposed variable with a new coordinate to estimate the continuous change in the bedtime variable for the effects on physical fitness variables. The converted bedtime variable ranged from −11 to 12. In the transposed variable, value 0 represents 00:00 or 12.00AM, where “−1” represents one backward hour and “+1” represents one forward hour from 12.00AM respectively. For example, “−1” represents 23:00 or 11.00PM and “1” represents 01:00 or 1.00AM. From Table 1, the median value of bedtime is “−1” which shows that the bedtime of most respondents is 23:00 or 11.00PM.

The results of spearman correlation analysis are shown in Table 2. It was found that age showed moderate negative correlation with sit-ups performance (r = −0.52) accompanied by significant *p*-value (<0.001). There was an approximately low negative correlation between sleep duration and bedtime with a correlation coefficient of −0.26, also accompanied by a significant *p*-value (<0.001). Almost negligible correlations existed between the variables, except for age and bedtime. Whether or not there were nonlinear correlations between these variables we would need to discuss further, using the MARS method.

The *t*-test results indicate that there were gender differences in the physical fitness indicators (BMI, sit-ups, sit-and-reach and CEI). Although the difference between gender groups was also significant for each sleep condition variable, the difference was minimal.

The MARS models were constructed by assuming the sleep conditions as independent variables and physical fitness indicators as dependent variables. As the gender variables had stronger influence on the four physical fitness indicators (see Table 3), we stratified the analysis by gender and generated different MARS models to explore the effects of daily sleep conditions on physical fitness performance. The results of the models for the four physical fitness indicators by gender division are shown in Table 4, Table 5, Table 6 and Table 7. Based on the assumptions of the MARS model, the estimated coefficient reflected the trend and degree of the influence for the independent variable on the dependent variable. The knot represented the node of influence trend or degree change. Both the segment estimation coefficients and the knot values generated in the MARS regression process were the estimation results under data fitting.

Table 4 presents the selected variables for BMI under gender division. For males, age, sleep quality, and sleep duration were associated with BMI. The selected variables for females were age, sleep duration, and bedtime. It can be seen that sleep duration is an important variable for both genders and 7-h sleep duration was the important estimated knot for BMI of both genders. For females, sleep duration less than or more than 7 h presented different influences on the increase in BMI, with estimated coefficients of 0.11 and 0.19, which entered the parentheses and multiplied the variables to show the positive influences. However, for males, there is no correlation between sleep duration and BMI when sleep duration is more than 7 h.

In Table 5, it was found that all independent variables were selected into the estimation equation for the sit-ups indicator under gender division analysis. From the table, it can be observed that for both genders, the lower the quality of sleep, the lower the performance of sit-ups, and the knot was level 2. Sleep duration of 7 h was also the estimated knot for sit-ups. Based on the estimated coefficients of knots in both genders, sit-ups performance decreased in different degrees with more than 7 h of sleep duration, and sleep duration more than 7 h had no correlation with sit-and-reach performance.

The sit-and-reach performances by gender are shown in Table 6; the selected variables for males are age, sleep quality, and sleep duration. For females, age and sleep duration were associated with sit-and-reach performance. Age and sleep duration are important variables for both genders. The age influence modes of male and female groups were different, and the knot positions also opened the gap. Similar results were found for BMI and sit-ups; for males, the estimated knot of sleep duration for sit-and-reach was also 7 h. When the sleep duration exceeded 7 h, the negative influence on the sit-and-reach performance became relatively higher, since the estimated coefficient changed from 0.30 to −0.51. Males continued to experience a decrease in sit-and-reach performance as sleep duration increased. For males, the estimated knot position of sleep duration was 5 h. Males experienced a decrease in sit-and-reach performance when sleep duration was less or more than 5 hours.

Table 7 depicts that the estimation results of CEI by gender. The CEI of females was associated with age and sleep quality variables. Age was a significant variable for CEI of both genders and age-influence modes were different for CEI performances between males and females. Sleep quality was associated with women’s CEI when the sleep quality was greater than level 2. Sleep duration was not correlated with CEI in men or women. Bedtime was associated with CEI performance of males, but the trend was less regular, with two knots.

In short, from Table 4, Table 5, Table 6 and Table 7, it can be observed that age was an important independent variable for all four physical fitness indicators. In the sleep conditions, sleep duration was the most important factor associated with the physical fitness indicators since it was the only sleep condition simultaneously correlated with BMI, sit-ups and sit-and-reach in both genders. Bedtime and sleep quality were only associated with sit-ups performance in both genders.

The detailed statistical verification results of the MARS model for the four physical fitness indicators, including BMI, sit-ups, sit-and-reach and CEI, are shown in Tables S1–S8 in the Supplementary Materials, respectively. The corresponding Figures S1–S8 intuitively show the trend and degree of influence between sleep conditions and four physical fitness performances. Referring to the Supplementary Materials, the coefficient estimates of the selected variables were all significant (*p* ≤ 0.01).

## 4. Discussion

This study successfully explored the effects of daily sleep conditions on the performances of the four core physical fitness indicators among Taiwanese adults using an effective MARS regression method. Sleep duration, sleep quality and bedtime were statistically significant influence factors on physical fitness performances with different levels. Gender and age were the important predictor variables for all four physical fitness indicators. As existing studies have only explored the relationship between sitting time, lifestyle habits, sedentary behavior and physical fitness performance [22,24,25], our findings can provide more information about association between lifestyle and physical fitness performance.

As gender may have different body mechanism-corresponding modes on behavioral habits and physical fitness [37,38,39,40], we applied the MARS model on gender division to explore the effects of daily sleep conditions on physical fitness performance. We found that the selected important variables for BMI, sit-and reach and CEI indictors of males are different from that of females. Only the selected significant factors of sit-ups are the same in both genders. Our findings show that gender division is an important factor when evaluating the impact of daily sleep conditions on physical fitness performance.

Within the three sleep conditions, we found that sleep duration is the most important factor, as it associated with three physical fitness indictors including BMI, sit-ups and sit-and-reach. Since the MARS model evaluated the effects of independent variables on dependent variables by using estimated important knot position and segment estimation coefficients of independent variables, we also found that 7-h sleep duration was an important estimated knot for influencing the BMI of both genders, sit-ups of both genders, and sit-and-reach of males based on the MARS regression models of this study. Sleep duration less than or more than 7 h presented different degrees of influence on the increase in the BMI indicator and the decrease in sit-ups and sit-and-reach performances. As many of existing studies pointed out that individuals who slept for ≤ 6−8 hours and ≥ 9 hours were associated with an increased risk of diseases and decreased health responses [29,41,42,43,44], our findings suggest a potential reference knot point of sleep duration that may influence performances of the four core physical fitness indicators among Taiwanese adults.

This study focused on evaluating the relationships between the three sleep conditions on the four core physical fitness indicators since we aimed at exploring the association between lifestyle and physical fitness indictors. There are many studies that have concentrated on the effects of physical fitness indicators on clinical issues, such as obesity, diabetes, hypertension, heart disease and other chronic diseases [45,46,47]. For example, many studies used BMI to characterize the level of obesity and showed that as the BMI value increases, the risk of obesity increases when using BMI to measure the obesity of Taiwan residents [48,49,50,51,52,53,54]. However, to the best knowledge of authors, there are few studies which have dealt with the association between lifestyle, physical fitness indictors and clinical issues, and no study has focused on exploring the relationship between sleep conditions, physical fitness indictors and clinical issues. This is considered to be the direction of our future study when more clinical data associated with the NPFES data are available. Moreover, this study only used the gender variable as the confounding variable to reduce the heterogeneity of the used survey data. As our results indicate that age was important for variables associated with all four physical fitness indicators, in future research studying the relationship between sleep conditions and physical fitness, solely the age variable and/or both the age and gender variables can be considered as confounding factors to alleviate the interference of sample heterogeneity on the results.

The research design of this study is similar to that of some of the existing studies that used regression models to evaluate the influence of various lifestyle habits on physical fitness using cross-sectional data through regression models [24,55]. Therefore, the limitations of this study were the same as those of the studies using cross-sectional data, in that we estimated the influence factors and speculated the possible effects, but this did not represent causal inference. In addition, it is noted that this study was based in part on data from the NPFES 2014 conducted by MOE-SA. NPFES 2014 was not designed to investigate sleep conditions only, and the survey did not use the Pittsburgh Sleep Quality Index to evaluate sleep conditions.

## 5. Conclusions

This study focused on assessing the relationships between the three sleep conditions including sleep quality, sleep duration and bedtime on the four core physical fitness indictors involving BMI, sit-up, sit-and-reach based on NPFES data and the MARS method. By considering gender division, the results reveal that all four daily sleep conditions are correlated with all three physical fitness indicators to different degrees. This study also suggests that 7-h sleep duration was a potential reference knot point of sleep duration that may influence performances of the core physical fitness indicators among Taiwanese adults.

## Figures and Tables

**Table 1:** Statistical description.

Variables	Age	SQ	SD	Bedtime	BMI	S-u	S-and-r	CEI	Gender	
Min.	23	1	4	-11	13.67	0	−4	11.78	Male	29,562(48%)
1st Qu.	31	1	6	−1	21.26	16	17	50	Female	32,532(52%)
Median	40	2	7	−1	23.46	24	25	55.56		
Mean	41.24	1.72	6.85	−0.41	23.88	23.20	24.73	57.32		
3rd Qu.	51	2	8	0	26.02	31	33	62.94		
Max.	64	4	13	12	68.13	80	70	155.17		

SQ: sleep quality; SD: sleep duration; BMI: body mass index; S-u: Sit-ups; S-and-r: Sit-and-reach; CEI: cardiorespiratory endurance index.

**Table 2:** Correlation analysis.

Control Variables		Age	SQ	SD	Bedtime	BMI	S-u	S-and-r	CEI
age	Corr.	1	−0.01	−0.07	−0.20	0.16	−0.52	−0.03	0.09
	Sig.(2-tailed)		0.002	<0.001	<0.001	<0.001	<0.001	<0.001	<0.001
SQ	Corr.	−0.01	1	−0.12	0.03	−0.04	−0.07	−0.01	−0.03
	Sig.(2-tailed)	0.002		<0.001	<0.001	<0.001	<0.001	0.05	<0.001
SD	Corr.	−0.07	−0.12	1	−0.26	−0.05	−0.01	−0.03	−0.00
	Sig.(2-tailed)	<0.001	<0.001		<0.001	<0.001	0.01	<0.001	0.9
bedtime	Corr.	−0.20	0.03	−0.26	1	−0.01	0.18	0.01	−0.06
	Sig.(2-tailed)	<0.001	<0.001	<0.001		0.4	<0.001	0.9	<0.001
BMI	Corr.	0.16	−0.04	−0.05	−0.01	1	−0.02	−0.10	-0.12
	Sig.(2-tailed)	<0.001	<0.001	<0.001	0.4		<0.001	<0.001	<0.001
S-u	Corr.	−0.52	−0.07	−0.01	0.18	−0.02	1	0.07	0.04
	Sig.(2-tailed)	<0.001	<0.001	0.01	<0.001	<0.001		<0.001	<0.001
S-and-r	Corr.	−0.03	−0.01	−0.03	0.01	−0.10	0.07	1	0.05
	Sig.(2-tailed)	<0.001	0.05	<0.001	0.9	<0.001	<0.001		<0.001
CEI	Corr.	0.09	−0.03	−0.00	−0.06	−0.12	0.04	0.05	1
	Sig.(2-tailed)	<0.001	<0.001	0.9	<0.001	<0.001	<0.001	<0.001	

SQ: sleep quality; SD: sleep duration; BMI: body mass index; S-u: Sit-ups; S-and-r: Sit-and-reach; CEI: cardiorespiratory endurance index.

**Table 3:** *T*-test analysis by gender.

Variables	Male (gender = 0)	Female (gender = 1)	*p*-Value
age	40.01	42.36	<0.001
SQ	1.65	1.78	<0.001
SD	6.82	6.87	<0.001
bedtime	−0.35	−0.46	<0.001
BMI	24.88	22.98	<0.001
S-u	28.86	18.06	<0.001
S-and-r	21.69	27.49	<0.001
CEI	58.16	56.56	<0.001

SQ: sleep quality; SD: sleep duration; BMI: body mass index; S-u: Sit-ups; S-and-r: Sit-and-reach; CEI: cardiorespiratory endurance index.

**Table 4:** Estimation results for BMI by gender.

BMI	Male		Female	
	Selected Variable	Coefficient Estimation	Selected Variable	Coefficient Estimation
Intercept		25.08		22.67
Age	max(0, age-35)	−0.01	max(0, age-38)	0.05
	max(0, 35-age)	−0.14	max(0, 38-age)	−0.08
Sleep quality	max(0, sleep_quality-2)	0.00	——	——
	max(0, 2-sleep_quality)	0.23	——	——
Sleep duration	max(0, sleep_duration-7)	0.00	max(0, sleep_duration-7)	0.11
	max(0, 7-sleep_duration)	0.32	max(0, 7-sleep_duration)	0.19
Bedtime	——	——	max(0, bedtime-(-1))	0.00
	——	——	max(0, -1-bedtime)	0.18

**Table 5:** Estimation results for sit-ups by gender.

Sit-ups	Male		Female	
	Selected Variable	Coefficient Estimation	Selected Variable	Coefficient Estimation
Intercept		29.30		22.65
Age	max(0, age-42)	−0.49	max(0, age-33)	−0.33
	max(0, 42-age)	0.43	max(0, 33-age)	0.63
			max(0, age-43)	−0.19
Sleep quality	max(0, sleep_quality-2)	−0.66	max(0, sleep_quality-2)	−0.91
	max(0, 2-sleep_quality)	0.57	max(0, 2-sleep_quality)	0.24
Sleep duration	max(0, sleep_duration-7)	−0.97	max(0, sleep_duration-7)	−0.74
	max(0, 7-sleep_duration)	0.00	max(0, 7-sleep_duration)	0.00
Bedtime	max(0, bedtime-1)	0.00	max(0, bedtime-0)	−0.07
	max(0, 1-bedtime)	−0.56	max(0, 0-bedtime)	−0.65

**Table 6:** Estimation results for sit-and-reach by gender.

Sit-and-reach	Male		Female	
	Selected Variable	Coefficient Estimation	Selected Variable	Coefficient Estimation
Intercept		21.74		27.20
Age	max(0, age-28)	−0.08	max(0, age-38)	0.07
	max(0, 28-age)	0.28	max(0, 38-age)	0.24
Sleep quality	max(0, sleep_quality-3)	0.00	——	——
	max(0, 3-sleep_quality)	0.54	——	——
Sleep duration	max(0, sleep_duration-7)	−0.51	max(0, sleep_duration-5)	−0.50
	max(0, 7-sleep_duration)	0.30	max(0, 5-sleep_duration)	−1.40
Bedtime	——	——	——	——

**Table 7:** Estimation results for CEI by gender.

CEI	Male		Female	
	Selected Variable	Coefficient Estimation	Selected Variable	Coefficient Estimation
Intercept		57.44		58.28
Age	max(0, age-30)	0.12	max(0, age-56)	−0.17
	max(0, 30-age)	0.31	max(0, 56-age)	−0.10
Sleep quality	——	——	max(0, sleep_quality-2)	−0.92
	——	——	max(0, 2-sleep_quality)	0.00
Sleep duration	——	——	——	——
Bedtime	max(0, bedtime-2)	0.66	——	——
	max(0, bedtime--2)	−0.62	——	——
	max(0, -2-bedtime)	−0.56	——	——

## Supplementary Materials

The following are available online at https://www.mdpi.com/1660-4601/17/6/1907/s1, Table S1: Estimation results of the MARS model for BMI—male, Table S2: Estimation results of the MARS model for BMI—female, Table S3: Estimation results of the MARS model for sit-ups—male, Table S4: Estimation results of the MARS model for sit-ups—female, Table S5: Estimation results of the MARS model for sit-and-reach-male, Table S6: Estimation results of the MARS model for sit-and-reach—female, Table S7: Estimation results of the MARS model for CEI—male, Table S8: Estimation results of the MARS model for CEI—female. Figure S1: Trend diagram of the sleep condition and BMI—male, Figure S2: Trend diagram of the sleep condition and BMI—female, Figure S3: Trend diagram of the sleep condition and sit-ups—male, Figure S4: Trend diagram of the sleep condition and sit-ups—female, Figure S5: Trend diagram of the sleep condition and sit-and-reach—male, Figure S6: Trend diagram of the sleep condition and sit-and-reach—female, Figure S7: Trend diagram of the sleep condition and CEI—male, Figure S8: Trend diagram of the sleep condition and CEI—female.
