# Supplementary material for: The Effects of Daily Sleep Condition on Performances of Physical Fitness among Taiwanese Adults: A Cross-Sectional Study
Source: Int J Environ Res Public Health. 2020 Mar 15;17(6):1907. doi: 10.3390/ijerph17061907 (PMC7143214; doi:10.3390/ijerph17061907)
Supplement: Supplementary file 1 [file ijerph-17-01907-s001.pdf]

International Journal of
Environmental Research and Public Health

MDPI

Supplementary Materials

Table S1. Estimation results of the MARS model for BMI- Male.

Male-BMI	Estimate	Std. Error	t value	Pr(>|t|)	Sig.
(Intercept)	25.080	0.045	553.403	<2e-16	***
max(0, age-35)	-0.014	0.003	-5.228	2e-16	***
max(0, 35-age)	-0.140	0.006	-23.203	<2e-16	***
max(0, 7-sleep_duration)	0.323	0.030	10.635	<2e-16	***
max(0, 2-sleep_quality)	0.227	0.040	5.625	1.87e-08	***

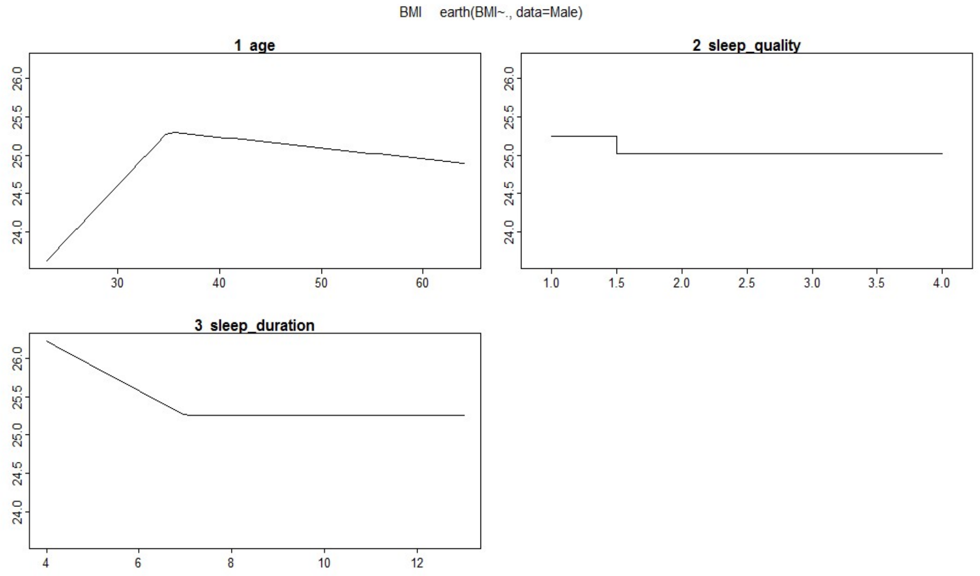

Figure S1. Trend diagram of the sleep condition and BMI- Male.

Table S2. Estimation results of the MARS model for BMI- Female.

Female-BMI	Estimate	Std. Error	t value	Pr(>|t|)	Sig.
(Intercept)	22.673	0.043	526.178	<2e-16	***
max(0, age-38)	0.051	0.003	18.001	<2e-16	***
max(0, 38-age)	-0.083	0.005	-16.857	<2e-16	***
max(0, -1-bedtime)	0.176	0.029	6.132	8.75e-10	***
max(0, sleep_duration-7)	0.106	0.036	2.937	0.00331	***
max(0, 7-sleep_duration)	0.192	0.032	6.077	1.24e-09	***

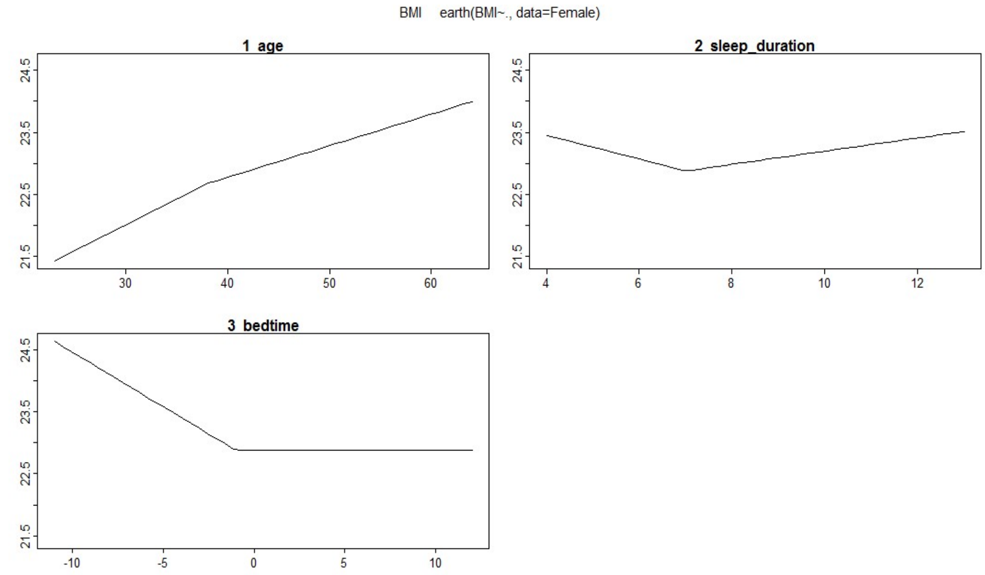

Figure S2. Trend diagram of the sleep condition and BMI-Female.

Table S3. Estimation results of the MARS model for sit-ups- Male.

Male-Sit-ups	Estimate	Std. Error	t value	Pr(>|t|)	Sig.
(Intercept)	29.303	0.142	206.342	<2e-16	***
max(0, age-42)	-0.494	0.010	-49.770	<2e-16	***
max(0, 42-age)	0.432	0.009	46.061	<2e-16	***
max(0, 1-bedtime)	-0.560	0.046	-12.261	<2e-16	***
max(0, sleep_duration-7)	-0.972	0.097	-9.991	<2e-16	***
max(0, sleep_quality-2)	-0.662	0.119	-5.577	2.47e-08	***
max(0, 2-sleep_quality)	0.571	0.111	5.125	2.99e-07	***

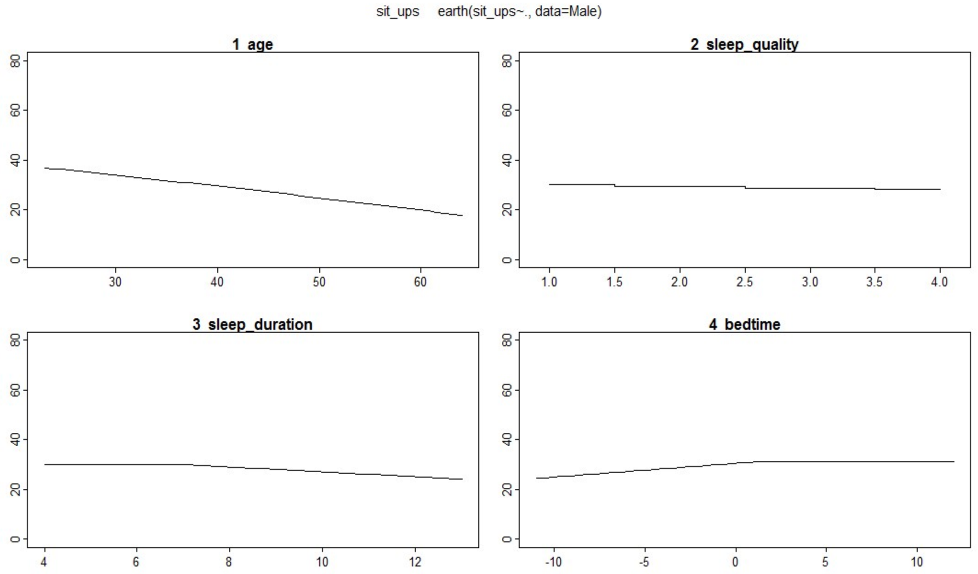

Figure S3. Trend diagram of the sleep condition and Sit-ups-Male.

Table S4. Estimation results of the MARS model for sit-ups-female.

Female-Sit-ups	Estimate	Std. Error	t value	Pr(>|t|)	Sig.
(Intercept)	22.653	0.150	150.791	<2e-16	***
max(0, age-33)	-0.332	0.019	-17.732	<2e-16	***
max(0, 33-age)	0.629	0.023	27.281	<2e-16	***
max(0, bedtime-0)	-0.073	0.025	-2.899	0.00375	**
max(0, 0-bedtime)	-0.651	0.053	-12.183	<2e-16	***
max(0, sleep_quality-2)	-0.909	0.097	-9.406	<2e-16	***
max(0, 2-sleep_quality)	0.238	0.107	2.223	0.02622	*
max(0, sleep_duration-7)	-0.743	0.085	-8.746	<2e-16	***
max(0, age-43)	-0.189	0.026	-7.297	3.02e-13	***

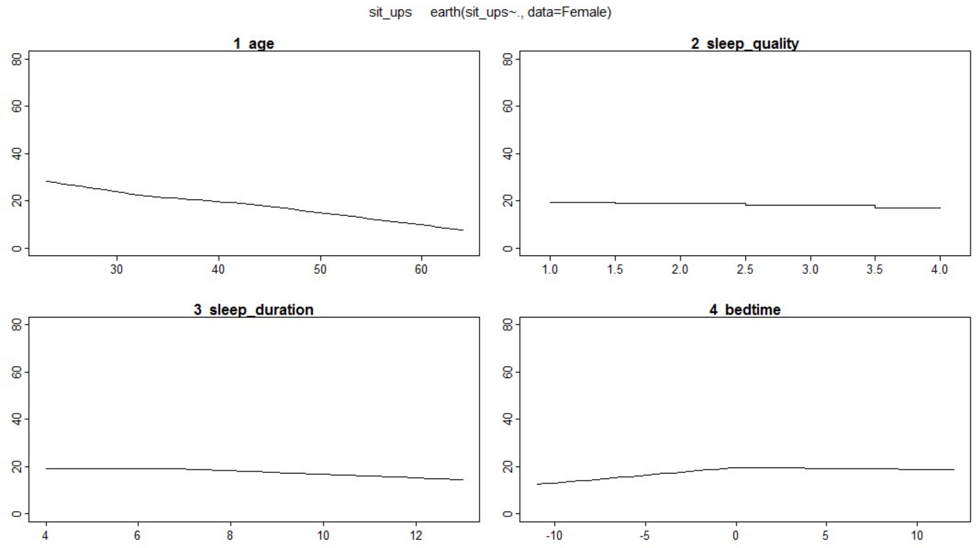

Figure S4. Trend diagram of the sleep condition and Sit-ups-Female.

Table S5. Estimation results of the MARS model for sit-and-reach-Male.

Male-Sit-ups	Estimate	Std. Error	t value	Pr(>|t|)	Sig.
(Intercept)	21.741	0.183	119.057	<2e-16	***
max(0, age-28)	-0.076	0.006	-11.831	<2e-16	***
max(0, 28-age)	0.281	0.050	5.594	2.24e-08	***
max(0, sleep_duration-7)	-0.507	0.123	-4.122	3.77e-05	***
max(0, 7-sleep_duration)	0.301	0.101	2.983	0.00285	**
max(0, 3-sleep_quality)	0.541	0.088	6.125	9.17e-10	***

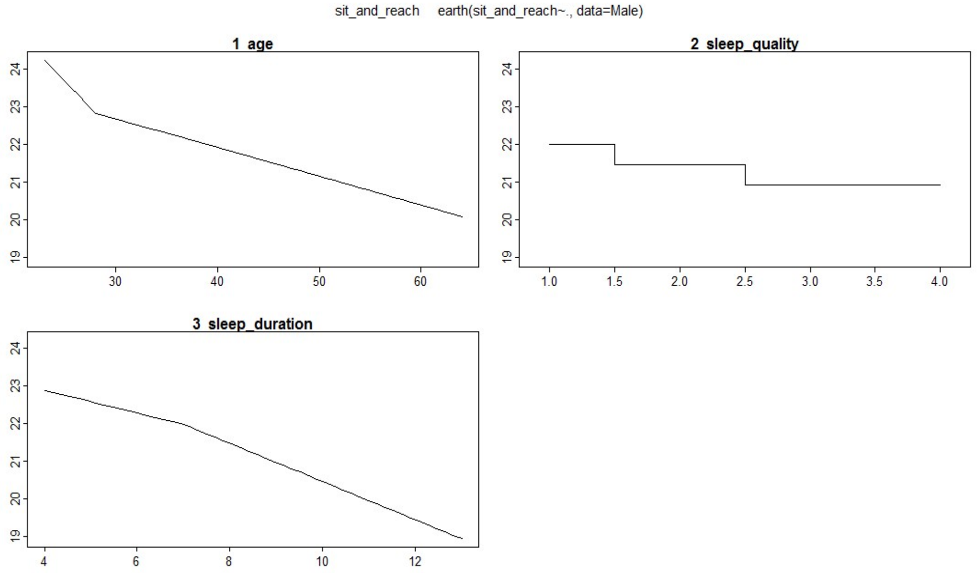

Figure S5. Trend diagram of the sleep condition and sit-and-reach-male.

Table S6. Estimation results of the MARS model for sit-and-reach- female.

Female-Sit-ups	Estimate	Std. Error	t value	Pr(>|t|)	Sig.
(Intercept)	27.196	0.168	161.706	<2e-16	***
max(0, age-38)	0.072	0.009	8.189	2.74e-16	***
max(0, 38-age)	0.236	0.015	15.376	<2e-16	***
max(0, sleep_duration-5)	-0.503	0.061	-8.207	2.35e-16	***
max(0, 5-sleep_duration)	-1.398	0.579	-2.414	0.0158	*

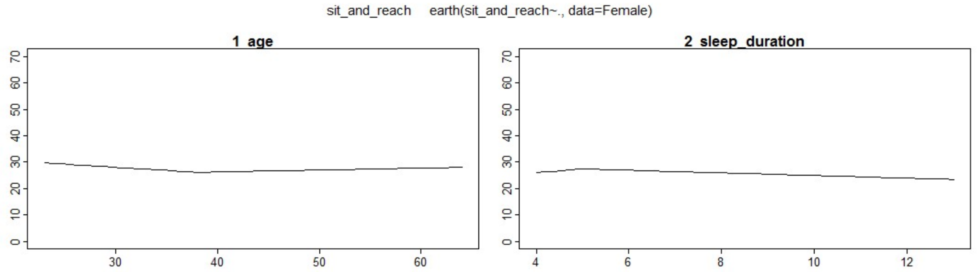

Figure S6. Trend diagram of the sleep condition and sit-and-reach-female.

Table S7. Estimation results of the MARS model for CEI- male.

Male-CEI	Estimate	Std. Error	t value	Pr(>|t|)	Sig.
(Intercept)	57.437	0.170	336.919	<2e-16	***
max(0, age-30)	0.121	0.008	15.567	<2e-16	***
max(0, 30-age)	0.309	0.038	8.179	2.97e-16	***
max(0, bedtime--2)	-0.617	0.070	-8.790	<2e-16	***
max(0, -2-bedtime)	-0.560	0.154	-3.630	0.000283	***
max(0, bedtime-2)	0.657	0.103	6.382	1.78e-10	***

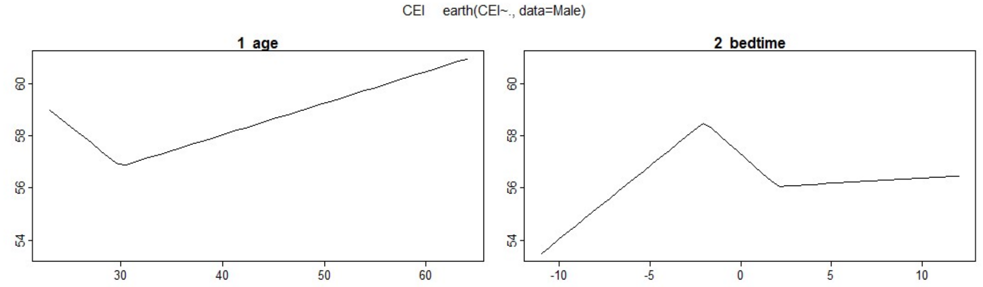

Figure S7. Trend diagram of the sleep condition and CEI- male.

Table S8. Estimation results of the MARS model for CEI- female.

Female-CEI	Estimate	Std. Error	t value	Pr(>|t|)	Sig.
(Intercept)	58.281	0.153	380.444	<2e-16	***
max(0, age-56)	-0.169	0.047	-3.621	0.000294	***
max(0, 56-age)	-0.097	0.008	-12.463	<2e-16	***
max(0, sleep_quality-2)	-0.918	0.134	-6.864	6.81e-12	***

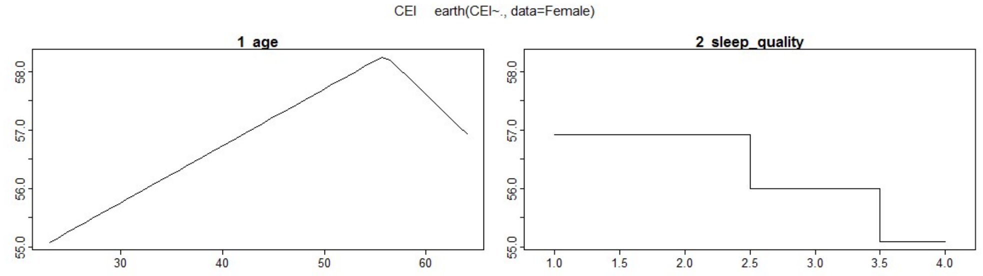

Figure S8. Trend diagram of the sleep condition and CEI-female.

.
